# Clinical audit of the management and outcomes of preterm pre-labour rupture of membranes at Aminu Kano Teaching Hospital

**DOI:** 10.11604/pamj.2022.43.41.32790

**Published:** 2022-09-26

**Authors:** Sulaiman Muhammad Daneji, Naimat Ibrahim Kasim, Idris Usman Takai

**Affiliations:** 1 Aminu Kano Teaching Hospital, Kano, Nigeria,; 2 Department of Obstetrics and Gynaecology, Faculty of Clinical Sciences, College of Health Sciences, Bayero University Kano, Kano, Nigeria

**Keywords:** Preterm pre-labour rupture of membranes, preterm premature rupture of the membranes, gestational age, Apgar score, prematurity

## Abstract

**Introduction:**

preterm pre-labour rupture of membranes (PPROM) is one of the important causes of preterm birth that can result in high perinatal morbidity and mortality along with maternal morbidity. The purpose of the study was to audit the management of women presenting with Preterm pre-labour rupture of membranes in Aminu Kano Teaching Hospital (AKTH).

**Methods:**

this was a retrospective audit on patients admitted with PPROM in AKTH over a period of 24 months. Data was analysed using SPSS version 22 and presented using percentages and compared with the audit standard. Chi-squared test was used to test for association (p-value <0.05).

**Results:**

the mean gestational age was 33.27±2.42 weeks. Diagnosis was made on all patients through history and clinical examination. Almost all patients received a course of erythromycin (88%), corticosteroid (84%) and magnesium sulphate (86%). Vaginal delivery was achieved in 57%. About 60% of the neonates were premature, 78% had Apgar score >7 at 5 mins, 50% were admitted in the special care baby unit and 72% survived. Chorioamnionitis and puerperal sepsis occurred in 8% and 21.7% of the mothers. Prolonged PPROM of >24 hours was statistically significantly associated with puerperal sepsis (χ^2^=7.218; p = 0.007) and perinatal mortality (χ^2^= 11.505, p = 0.001).

**Conclusion:**

despite high fidelity to institutional clinical practice guidelines in Aminu Kano Teaching Hospital there seems to be poor maternal and neonatal outcome with high perinatal mortality. Thus the guidelines need to be reviewed in context of improving the outcome.

## Introduction

Preterm pre-labour rupture of membranes (PPROM) is the loss of amniotic fluid before the onset of labour in pregnancies after foetal viability (>28 weeks of gestation) but before 37 weeks of gestation [1-3]. It occurs in 5 to 10% of all pregnancies [1,2]. It causes about 20% and 21.4% of prenatal mortalities and morbidity respectively [2]. About 32 to 40% of preterm deliveries are associated with preterm PROM, with 60 to 80% of delivering within 48 hours [1]. It is reported that black women have a higher risk for preterm PROM [3,4]. In Nigeria, the incidence of Preterm PROM was found to be 3.3%, 6.3% and 23.7% in Enugu, Calabar and Sokoto, while a prevalence of 0.9% was reported in Kano [5-8].

Preterm pre-labour rupture of membranes is classified into early (28-33^+6^ weeks) and late (34-36^+^^6^ weeks) [9,10]. The exact cause of preterm PROM is not known but risk factors attributed to it includes low socioeconomic conditions, smoking, multiple gestations, polyhydramnios, gestational hypertension and diabetes mellitus [9-12]. The diagnosis requires a thorough history, physical examination, and selected laboratory studies. This includes history of fluid drainage through the vagina. A speculum examination is performed but a digital cervical examination is avoided as it was shown to be associated with shortening of the latent period, increase morbidity and mortality [12-14]. Several factors have to be considered when managing patients with PPROM, it has to be individualised as its management can be challenging and controversial [15]. These include gestational age, availability of neonatal intensive care, presence or absence of maternal/foetal infection, presence or absence of labour, foetal presentation, foetal heart rate (FHR) tracing pattern, likelihood of foetal lung maturity, and cervical status (by visual) inspection unless induction is planned or the patient is in labour [13,16,17].

Women who have no contraindications to continuing the pregnancy should be offered expectant management until 37+0 weeks. Expectant management is associated with a risk of ascending infection and umbilical cord compression at all gestational ages, therefore this should be balanced against the risks of immediate delivery [17,18]. About half of the women who present with preterm PROM will go into labour within 24-48 hours and about 70-90% within 7 days [18,19]. The median latency after PPROM is 7 days and tends to shorten as the gestational age at PPROM advances.

Premature delivery can occur therefore a level III Nursery should be available for patients with PPROM [17]. The signs of clinical chorioamnionitis and some investigations, including full blood count and C-reactive protein, high vaginal swab, cardiotocography, biophysical profile score and Doppler velocimetry can be offered, but these tests are of limited value in predicting foetal infection [17]. Antibiotic therapy can reduce maternal and neonatal morbidity and also prolongs the latency period [17,20]. Antenatal corticosteroid therapy between 28 and 34 weeks of gestation is also thought to reduce neonatal morbidity by promoting foetal lung maturity [17,18,20]. The use of Magnesium sulphate in less than 32 weeks is also recommended for neuro protection especially those at risk of imminent delivery [18,20]. At 34 weeks, the risk of continued expectant management begins to exceed the risk of prematurity and therefore delivery is generally recommended by ACOG [18].

Many studies were done on PROM with no much specification on preterm PROM whereas it is one of the important causes of preterm birth that can result in high perinatal morbidity and mortality along with maternal morbidity. This study aims to audit the management of Preterm PROM in Aminu Kano Teaching Hospital. It will give us a guide on how PPROM is managed in the hospital. This will improve care and will decrease the morbidity and mortality associated with PPROM.

## Methods

The audit was a retrospective study from the case folders of patients managed for preterm PROM from 1^st^ January 2018 to 31^st^ December, 2019. A structured proforma was used to extract information from the folders which included sociodemographic characteristics, obstetric history, clinical examination findings, routine investigations, mode of delivery, perinatal/neonatal information such as birth weight, Apgar score and foetal outcome. Maternal complications that were assessed included chorioamnionitis and puerperal sepsis.

**Audit standard/clinical practice guidelines for management of PPROM at AKTH:** i) diagnosis of preterm rupture of membranes is made from maternal history, followed by a sterile speculum examination; ii) during the sterile speculum examination one looks for pooling of fluid in the posterior fornix, trickling of fluid from the cervical os and presence of cord; iii) investigations including full blood count, high vaginal swab and ultra sound scan, cardiotocograph are carried out, to determine the urgency of management; iv) following the diagnosis of preterm prelabour rupture of membranes (PPROM) an antibiotic (preferably erythromycin) should be given for 10 days or until the woman is in established labour (whichever sooner); v) women who have PPROM between 28 and 34 weeks should be offered corticosteroids; vi) a combination of clinical assessment, maternal blood tests (C-reactive protein and white cell count) and foetal heart rate should be used to diagnose chorioamnionitis in women with PPROM, these parameters should not be used in isolation; vii) in women who have PPROM and are in established labour or having a planned preterm birth within 24 hours, intravenous magnesium sulphate should be offered between 28 and 34 weeks of gestation for neuroprotection and tocolysis to allow some time for the corticosteroids to act.

### Definitions

**Extremely low birth weight (ELBW):** is neonatal birth weight within 24 hours of delivery less than 1000 grams.

**Very low birth weight (VLBW):** is neonatal birth weight within 24 hours of delivery between 1000 grams and 1499 grams.

**Low birth weight: neonatal birth weight (LBW):** within 24 hours of delivery between 1500 to 2499 grams.

**Normal birth weight:** neonatal birth weight within 24 hours of delivery more than 2500 grams [21].

**Birth asphyxia (perinatal asphyxia):** is defined based on the Apgar scoring as the 5^th^ min Apgar score of <7 and the 1^st^ min Apgar score of <7 [22].

**Prematurity:** classification into premature neonates or mature neonates was done using the Dubowitz and Ballard scoring charts in the special care baby unit of the hospital (SCBU). Dubowitz method utilizes 21 combined criteria including external score and neurological score, while the Ballard score utilises 12 combined criteria including physical score and neuromuscular score [23].

**Inclusion criteria:** all pregnant women with spontaneous rupture of membrane between gestational age of 28 and 36 weeks 6 days before the onset of labour.

**Exclusion criteria:** women whose membranes were ruptured but have the following additional complications like antepartum haemorrhage, severe preeclampsia/eclampsia, multiple gestation, intrauterine foetal death, foetal congenital anomaly or were admitted in established labour.

Data was entered into Microsoft excel sheet and was transferred to SPSS version 22 for analysis. Frequency and percentages of variables were determined and put in tables. The association between the period after PPROM, divided into <24 hours and >24 hours, was compared with maternal morbidity (puerperal sepsis, chorioamnonitis) and perinatal morbidity (Apgar score, neonatal sepsis and perinatal mortality) variables using Chi-squared test. Significant values were set at p-value of <0.05.

## Results

A total of 6515 deliveries were recorded during the study period. There were 92 cases of preterm pre-labour rupture of foetal membranes giving a prevalence of 1.4%. Sixty (65.2%) case notes retrieved from the medical records department were analysed. The mean age of the patients was 27.65±5.36 years and ranged from 19 to 39 years. Eighty-five percent reside in urban area and majority of them (65%) were unemployed. The mean gestational age was 33.27±2.42 weeks and ranged from 28 weeks to 36 weeks plus 6 days. Fifty-one percent of the study population were booked and the mean number of antenatal visit among them was 3.19±1.05. Only 24 (40%) presented within 24 hours of onset of PPROM as it is depicted in Table 1.

**Table 1:** socio-demographic and obstetric characteristics of patients with PPROM

Variables	Frequency	Percentage
**Age (yr)**		
<20	1	1.7
20-24	15	25.0
25-29	24	40.0
30-34	11	18.3
35-39	9	15.0
**Religion**		
Islam	47	78.3
Christianity	13	21.7
**Education**		
None	2	3.3
Primary	12	20.0
Secondary	23	38.3
Tertiary	23	38.3
**Ethnicity**		
Hausa	39	65.0
Fulani	4	6.7
Yoruba	3	5.0
Igbo	3	5.0
Others	11	18.3
**Marital status**		
Single	2	3.3
Married	58	96.7
**Residence**		
Urban	51	85.0
Rural	9	15.0
**Employment status**		
Yes	21	35.0
No	39	65.0
**Gestational age (wks)**		
28^-0^ - 31^+6^	14	23.3
32 - 33^+6^	23	38.3
34 - 36^-6^	23	38.3
**Parity**		
Nullipara	19	31.7
1-4	34	56.7
≥5	7	11.7
**Period of presentation**		
Within 24 hr	24	40.0
Above 24 hr	36	60.0
**Abdominal pain**		
Yes	16	26.7
No	44	73.3
**Presentation**		
Cephalic	50	83.3
Others	10	16.7

All patients had documented history of spontaneous rupture of membranes. Speculum examination was performed on all 60 cases, ultrasound scan done in 88.3% of the cases (Table 2). Less than 2% of the population presented with thick meconium; 6.7% had offensive liquor smell. Digital examination was avoided in 91.7% of the patients. Ultrasound scan was done in 88% of cases to help in confirming the diagnosis; however, cardiotocograph was used to monitor foetal heart rate in only 42% of the patients. Full blood count was done in all 60 cases, and 57 (95%) received an antibiotic (erythromycin in 88% of patients). Corticosteroid was given to 31 patients (83.8%) of those with gestational age of less than 34 weeks, 12 patients (85.7%) of those with gestational age of less than 32 weeks received MgSO_4_. Tocolysis in the form of oral Nifedipine 20mg daily was administered in only 5 (8.3%). Mode of delivery was by spontaneous vaginal delivery in 34 (56.7%) cases and 26 (43.3%) cases by caesarean section.

**Table 2:** frequencies and percentages of standard achieved

Criteria	Frequency	Percentage achieved (%)
1. The diagnosis of spontaneous rupture of membranes by maternal history	60	100
2. Followed by a sterile speculum examination	60	100
3. Presence of amniotic fluid in the posterior fornix	60	100
4. Digital examination should be avoided	55	91.7
5. Ultrasound examination in some cases to help confirm diagnosis	53	88.3
6. Women observed for signs of chorioamnionitis. At least 12 hourly clinical assessment: foetal heart rate	57	95
7. Weekly high vaginal swabs	16	26.7
8. At least weekly maternal FBC	57	95
9. Foetal monitoring using CTG should be done	25	41.7
10. Antibiotics preferably erythromycin administered	53	88.3
11. Administer corticosteriod in women with GA less than 34 weeks	31	83.8
12. A magnesium sulphate in women with GA less than or equal to 32 weeks	12	85.7
13. Delivery should be considered between 34 weeks-36 weeks	39	65

Thirteen (21.7%) patients had puerperal sepsis, 8.3% had postpartum haemorrhage and 8.3% had chorioamnionitis. Thirty-five percent (35%) of the women delivered within 48 hrs of admission while two (3.3%) delivered after 7 days of admission. Two (3.3%) babies had extreme low birth weight, and 25 (41.7%) babies weighed above 2.5kg. The major morbidity in the neonates was due to sepsis in 16 (26.7%) followed by birth asphyxia in 14 (23.3%). Thirty-one (51.7%) were admitted in SCBU and about 60% of the neonates delivered were classified as being premature using the Dubowitz and Ballard scoring system.

In this study perinatal mortality was found to be 28.3%. Perinatal mortality was not statistically significantly associated with maternal age and parity (Table 3). However, there was a statistically significant association with gestational age in which more perinatal death was found in those with gestational ages below 34 weeks (76.5% of the deaths occurred). There is also a relationship with antenatal care, about 70% of the death also occurred in those that were unbooked (χ^2^= 4.705, p = 0.029). Duration of presentation after PPROM is statistically associated with perinatal death as more death (94.1%) occurred among those that presented after 24 hours (χ^2^= 11.505, p = <0.001). Perinatal death was also statistically associated with SCBU admission as 88% of the death occurred amongst them (χ^2^= 12.703, p = <0.001). Chorioamnionitis is also statistically related to perinatal death (χ^2^= 7.171, p = 0.020).

**Table 3:** association between perinatal mortality with some patients’ characteristics

Variable	Foetal outcome		χ2	p-value
	Dead	Alive		
	N=17 (n(%)	N=43 (n(%)		
**Maternal age**				
<20	1(6.3)	0(0)	Fishers exact (3.947)	0.426
20-24	5(31.3)	10(23.8)		
25-29	7(43.8)	17(40.5)		
30-34	2(12.5)	12(28.6)		
35-39	1(6.3)	3(7.1)		
**Parity**				
Nullipara	7(41.2)	12(27.9)	1.416	0.460
1-4	9(52.9)	25(58.1)		
≥5	1(5.9)	6(14.0)		
**Gestational age (wks)**				
28-0 - 31^+6^	7(41.2)	7(16.3)	Fishers exact (7.279)	0.025
32 - 33^+6^	6(35.3)	10(23.3)		
34 - 36^-6^	4(23.5)	26(50.5)		
**ANC status**				
Booked	5(29.4)	26(60.5)	4.705	0.029
Unbooked	12(70.6)	17(39.5)		
**Period of presentation**				
Within 24 hr	1(5.9)	23(53.5)	11.505	<0.001
Above 24 hrs	16(94.1)	20(46.5)		
**SCBU admission**				
Yes	15(88.2)	16(37.2)	12.703	<0.001
No	2(11.8)	27(62.8)		
**Chorioamnionitis**				
Yes	4(23.5)	1(2.3)	7.171	0.020
No	13(76.5)	42(97.7)		

df=1

Women with PPROM longer than 24 hours were more likely to have puerperal sepsis at 33.3% compared with 4.2% of women with less than 24 hours (Table 4). This difference was statistically significant (χ^2^= 7.218; p = 0.007). PPROM duration of >24 hours is associated with Apgar score at 5 minutes <7 (30.6%) compared with 12.5% of women with PPROM of <24 hours though this difference, was not statistically significant (χ^2^= 2.624; p = 0.105). Women with PPROM of >24 hours, 11.1% had chorioamnionitis compared with 4.2% in women with PPROM of <24 hours (χ^2^=0.909, p=0.340).

**Table 4:** association between PPROM presentation duration with maternal and neonatal morbidity

Variable	Period of presentation		χ2	p-value
	<24 hours	>24 hours		
	N=24 n(%)	N=36 n(%)		
**APGAR Score**			2.624	0.105
<7	3(12.5)	11(30.6)		
≥7	21(87.5	25(69.4)		
**Peuperal sepsis**			7.218	0.007
Yes	1(4.2)	12(33.3)		
No	23(95.8)	24(66.7)		
**Chorioamnionitis**			0.909	0.340
Yes	1(4.2)	4(11.1)		
No	22(95.8)	32(88.9)		
**Neonatal sepsis**			6.875	0.009
Yes	2(8.3)	2(8.3)		
No	22(91.7)	22(91.7)		
**Perinatal mortality**			11.505	0.001
Yes	1(4.2)	16(44.4)		
No	23(95.8)	20(55.6)		

df=1

## Discussion

The prevalence of PPROM in this study was 1.4% which is higher than 0.9% that was reported in a previous a study in Kano [8], lower than the 3.3% reported from Enugu PPROM [5] and 5.7% reported from Ile-Ife [24] a higher prevalence of 13.7% in a study in Tabor Ethiopia [25]. The high prevalence of PPROM in this study when compared to what was reported in the same centre in 2017 might be explained by the availability of free antenatal and delivery services in the state government owned facility. Thus, all presumed normal deliveries would have presented their living those with complications of pregnancy to present to the study site which is a fee paying centre and more expertise are present as shown that close to half of the patients were unbooked when compared to the previous study. This study is a retrospective study which may not include patients who went into spontaneous labour following a short latency period and some patients may not present till when they are in established labour might be the explanation for the lower prevalence in this study. The mean age of the patients is 27.7 years and the highest number of patients was in the 25-29 age group 24 (40%) which is similar to 26-30 years age range reported by Okeke *et al*. in Enugu [5] and the mean age of 27.5 in a Brazilian study this is likely due to this age group being more sexually active and are at higher risk of sexually transmitted infections.

The results of this audit showed that the diagnosis of PPROM from history, sterile examination and documentation of presence amniotic fluid in the posterior fornix was fully achieved. This is similar to the audit in Royal Women's Hospital, Melbourne, Australia [26]. Digital examination was avoided in 91.7% of the patients as recommended by the Royal College of Obstetricians and Gynaecologists (RCOG) and other studies as it is documented to decrease latency period [12-14,20]. Ultrasound scan was done in 88.3% of patients though this was not to confirm the diagnosis of PPROM only but to ascertain the gestational age, amniotic fluid index and foetal presentation. The RCOG advices that demonstration of oligohydramnios may be useful to support the clinical diagnosis of PPROM [20]. Clinical monitoring for signs chorioamnionitis was limited to foetal heart rate monitoring and was accomplished in 95% of the patients, however cardiotocograph was used to monitor foetal heart rate in only 42% of the patients as it is not readily available to be used on all patients, other tools for clinical monitoring include maternal temperature and pulse rate [26,27]. High vaginal swabs were taken at presentation for 27% of the patients this is lower than what was reported at Melbourne women hospital because in most instances the results may not be out when most of the patients have delivered so physicians may feel reluctant to take the samples [26]. We found that all patients managed in this hospital were given at least a dose an antibiotic which is similar to what was obtained in previous audits from Royal Women Hospital Melbourne, Australia and Sohag University Hospital, Egypt [26,27]. The antibiotic administered was erythromycin where about 88.3% received the drug as recommended by the RCOG and adapted by the department [20]. Thirty seven patients had PPROM at less than 34 weeks out of which 83.8% of them received corticosteroids and 85.7% of those with gestational ages of less than 32 weeks received magnesium sulphate for neuroprotection as adapted in the department following recommendations by RCOG [20,28]. Previous audit in Australia and Egypt showed similar results [26,27].

In this study the presence of at least one maternal morbidity was 21.7% which is similar to study in Enugu in Nigeria (20%) [5]. Perinatal mortality was found to be 28.3% which is higher than 8.9% reported from Enugu in Nigeria, 6.8% reported by Kayiga *et al*. in Uganda and perinatal mortality of 10% reported from India [5,29,30]. These discordant findings highlight the variability of practice between different clinicians belonging to different region of the world and the need for further research and consensus guidelines in managing this common presentation. Factors which might contribute despite effective use of antibiotics and corticosteroids could be lack of uninterrupted power supply in the special care baby unit coupled with lack of enough manpower to take of the neonates as most of the neonates were delivered alive. Those with good Apgar score at 5 mins were 77% and those admitted in the SCBU were 52% amongst which about 50% died. The issue of adulterated drugs in our environment which is very common in developing countries especially in recent years due to economic hardship could also contribute with poverty, illiteracy and poor health seeking behaviour. There was apparent lack of differential impact of duration of PPROM on both Apgar scores of the newborns and the prevalence of chorioamnionitis in this review.

## Conclusion

This audit has highlighted a number of aspects of the clinical practice guideline that can be improved, it has shown that for majority of admissions the basic principles are being followed (consistent with the clinical practice guidelines as adapted from RCOG Green-top guidelines) [20]. Fidelity to current practice guidelines at AKTH did not translate to optimal or desirable perinatal and maternal outcomes based on evidence provided by the clinical audit. Consequently, the clinical guidelines would need to be re-assessed by a multidisciplinary team to identify gaps or areas that require revamping. Additionally, monitoring and continuous evaluation of policy and practice of PPROM management should be implemented to improve efficiency, quality of care, health outcomes and patient satisfaction in AKTH.

**Recommendations:** based on the review of literature and retrospective audit of management of women admitted in AKTH with PPROM a number of recommendations can be made: 1) Further studies and subsequent cyclical clinical audits to assess for quality improvements and clinical governance should be made especially with addition of auditing the management in the SCBU. 2) Appropriate documentation of performed ultrasound results in the folders of patients. 3) Mandatory high vaginal swabs for all women with PPROM so appropriate antibiotic therapy is instituted following culture and sensitivity results of amniotic fluid and /or endocervical swabs to avoid drug resistance. 4) An antibiotic algorithm that specifies antibiotics (dose, frequency and route of administration) in suspected intrauterine sepsis should be made easily accessible to staffs.

### What is known about this topic

- A significant number of preterm deliveries are associated with preterm PROM;
- Antibiotic therapy reduces maternal and neonatal morbidity.

### What this study adds

- High level of adherence to clinical management guidelines in obstetrics, is feasible and attainable in tertiary health facilities in low- and middle-income countries;
- Prevention of perinatal and maternal infections associated with PPROM requires more than the routine use of generic, guideline-directed antibiotics; local antibiogram-based therapy may be more effective for management.
